# Healthcare Professionals’ Experiences of Telemedicine Supporting Outpatient Endometriosis Care: A Qualitative Study of Tele-Patient-Reported Outcome Measures

**DOI:** 10.3390/ijerph23050671

**Published:** 2026-05-19

**Authors:** Maria M. Feenstra, Anne Sidenius, Charlotte Nielsen, Martin Rudnicki

**Affiliations:** 1 Department of Gynaecology and Obstetrics, Odense University Hospital, 5000 Odense, Denmark; 2 Research Unit of Gynaecology and Obstetrics, Department of Clinical Research, Faculty of Health Sciences, University of Southern Denmark, 5000 Odense, Denmark; 3 Department of Gynaecology and Obstetrics, Kolding Hospital, University Hospital of Southern Denmark, 6000 Kolding, Denmark; 4 Department of Prevention, Health Promotion & Community Care, Copenhagen University Hospital–Steno Diabetes Center Copenhagen, 2730 Herlev, Denmark; anne.sidenius@regionh.dk; 5 Research Unit for Plastic Surgery, Odense University Hospital, 5000 Odense, Denmark; charlotte.nielsen@rsyd.dk; 6 Department of Oral and Maxillofacial Surgery, Odense University Hospital, 5000 Odense, Denmark; 7 Department of Clinical Research, Faculty of Health Sciences, University of Southern Denmark, 5230 Odense, Denmark

**Keywords:** telemedicine, patient-reported outcome measures, text message, video consultation, endometriosis, focus group, interpretive description, triage, outpatient follow-up, chat

## Abstract

**Highlights:**

**Public health relevance—How does this work relate to a public health issue?**

- Endometriosis is estimated to affect one in ten women worldwide.
- Women with endometriosis often do not receive adequate support and treatment tailored to their needs.

**Public health significance—Why is this work significant to public health?**

- Telemedicine may improve endometriosis care; however, few telemedicine initiatives have been integrated into outpatient follow-up care.
- This study explores healthcare professionals’ experiences with an endometriosis care model supported by telemedicine.

**Public health implications—What are the key implications or messages for practitioners, policymakers, and/or researchers in public health?**

- Telemedicine in endometriosis outpatient follow-up supports patient triage and provides healthcare professionals with flexible consultation options, which may help reduce unnecessary patient visits.
- Text message communication proved to be an important element in patient triage.
- Providing healthcare professionals with continuous communication and technical training during the digital transformation of healthcare is essential. However, maintaining organisational resilience may also require restructuring technical support services, including the provision of on-site assistance in clinical practice.

**Abstract:**

Background: Telemedicine may advance endometriosis care, but few initiatives are integrated in outpatient follow-up. A novel telemedicine approach—tele-patient-reported outcome measures (telePROM)—includes an endometriosis-specific questionnaire and phone and video consultations combined with text messaging (chat) with a multidisciplinary endometriosis team. This study explores how healthcare professionals experience telePROM and its integration in clinical practice. Methods: A qualitative study guided by interpretive description methodology. Data were generated through observations and focus group interviews conducted between January 2023 and March 2024 at a referral centre for endometriosis within a university hospital. A purposive sample of ten healthcare professionals comprising physicians, nurses and a medical secretary participated in the focus group interviews. Inductive analysis was inspired by interpretive description and carried out through an iterative process involving four steps, leading to the development of final themes and interpretation. Results: Three themes were identified from analysis: (1) Balancing Personalised Care With Increased Clinical Complexity; (2) Changing Professional Boundaries in a Digitally Supported Care Model; and (3) System Friction and Flexibility when Integrating TelePROM. Conclusions: Telemedicine improved endometriosis care by supporting patient-initiated and personalised consultations. However, sustainable, effective, and safe integration of telemedicine appears to require clinical experience, interdisciplinary collaboration, and supervision. Text communication (chat) proved to be an important element to ensure collection of additional information to complement patient-reported outcomes and it is essential for patient triage; yet it is rarely described in the literature. Ensuring organisational resilience during the digital transformation of healthcare requires ongoing training of healthcare professionals’ communicative and digital competences and may necessitate restructured technical support, including designated telemedicine experts in clinical practice to eliminate technical disruptions. These initiatives may contribute to and support the future implementation of telemedicine in healthcare.

## 1. Introduction

Up to 10% of women in their reproductive age are affected by endometriosis, a painful chronic disease [1,2,3] estimated to affect 190 million women worldwide [4] and for which treatment options remain limited. No cure exists, and hormonal treatment and/or surgery, often combined with painkillers, therefore aims to reduce the symptom burden of endometriosis and improve patients’ daily lives [5,6]. However, severe symptoms of the disease can lead to significant consequences for patients’ physical and mental health [7,8], and their symptom burden determines the need for follow-up, resulting in long-term monitoring and follow-up care.

Telemedicine has been shown to enhance the management and monitoring of patients with chronic diseases [9,10], further emphasising its potential to enable more holistic and person-centred endometriosis care [11,12,13]. However, there has been little integration of telemedicine initiatives in routine outpatient follow-up of endometriosis [11,14,15,16]. Digital care interventions in endometriosis have been developed, e.g., a self-administered mindfulness- and acceptance-based self-management online programme named ‘MY-ENDO’, virtual mindfulness-based therapy provided by a social worker, the ‘Endo-App’ prescribed by pain specialists, and a supportive text message programme named ‘EndoSMS’ [17,18,19,20,21]. However, these are designed as supplements to specialised endometriosis treatment and not integrated in the follow-up care pathway in outpatient gynaecology. Moreover, 48 patient-reported outcome measures have been identified for use in routine endometriosis care, capturing different dimensions of the disease’s impact on patients. Still, consensus regarding which outcome measures are relevant in outpatient care is yet to be established and developed with attention to, for example, the relevance, length and administration mode [22].

Telemedicine is defined by WHO as the delivery of healthcare services using information and communication technologies, when healthcare professionals and patients are separated by distance [23]. In Denmark, a novel telemedicine-supported follow-up approach was developed for routine clinical care in endometriosis: tele-patient-reported outcome measures (telePROM). TelePROM consists of an endometriosis-specific questionnaire that enables patients to self-report symptoms and well-being before a follow-up consultation; also defined as a patient-reported outcome measure, further supporting patient triage. TelePROM also includes telephone and video consultations and introduces nurse-led consultations in combination with asynchronous text message communication (chat) with an endometriosis care team. Patients referred to the endometriosis outpatient clinic can access telePROM elements through the ‘Mit Sygehus’ (My Hospital) app or web browsers (https://mit.rsyd.dk, accessed on 13 May 2026), with the only requirements being possession of a personal identifier (MitID) and Danish language proficiency. Patients’ experiences of telePROM have already been explored and were mainly positive. However, an unmet need among patients was identified: the provision of an educational programme in telePROM and endometriosis, as well as technical support [24].

Studies have previously identified important facilitators and barriers to the implementation of telemedicine in chronic diseases such as diabetes and chronic heart failure [10,25], as well as among healthcare professionals [26]. However, differences related to specific diseases and contexts do exist, highlighting the importance of investigating user experiences. To gain insight into the application of telePROM and further improve the adoption of telemedicine in endometriosis care, this study aims to investigate how healthcare professionals comprising physicians, nurses and medical secretaries experience outpatient follow-up of endometriosis when supported by telePROM. Particular attention was given to exploring healthcare professionals’ experiences of integrating telePROM into their routine clinical practice.

## 2. Methods

### 2.1. Design

We conducted a qualitative study informed by the methodology of interpretive description. The application-oriented approach in interpretive description seeks to offer insights into clinical problems rooted in healthcare and provide knowledge to guide, develop, and improve clinical practice [27]. TelePROM is a complex healthcare intervention necessitating organisational changes during implementation, such as the introduction of a new patient flow and new work procedures when integrated into outpatient follow-up between healthcare professionals and patients. The methodology of interpretive description ensures flexibility in terms of methods, inclusion criteria, and analysis, and it provides applicable directions for clinical practice [28]. To ensure quality and transparency, the Consolidated Criteria for Reporting Qualitative Research guided the reporting of the results [29].

### 2.2. Setting and Intervention

TelePROM was developed and implemented at a referral centre for endometriosis within a Danish university hospital. Patients residing in the second-largest region of Denmark who are diagnosed with (or suspected of having) endometriosis are referred to this clinic, resulting in travel times of up to four hours per visit for many.

**Figure 1:**
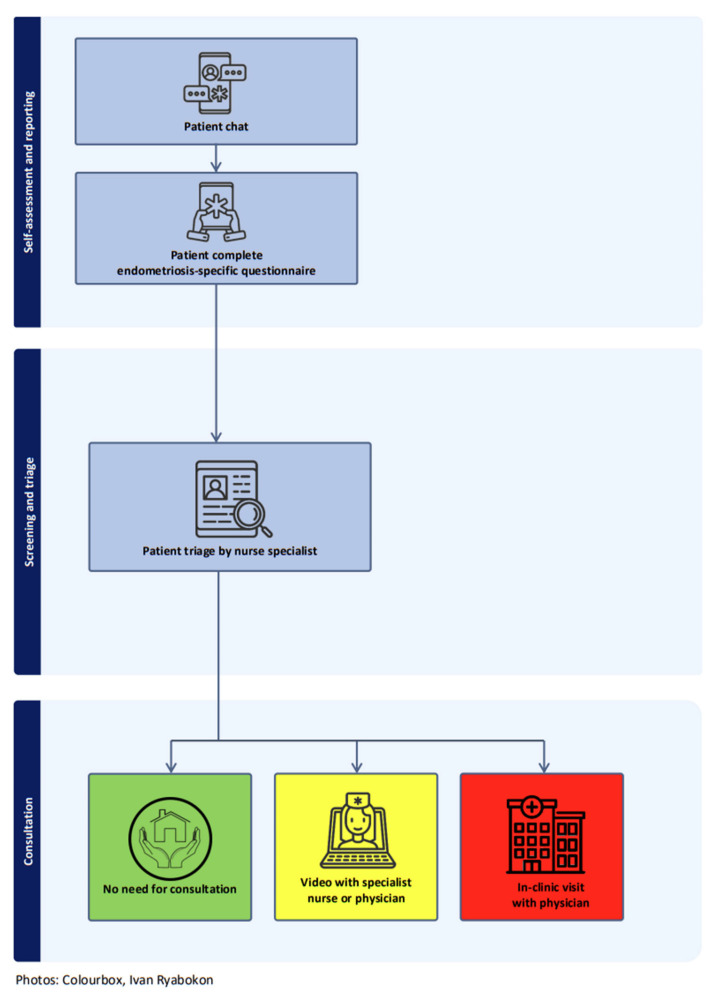
Flowchart of the telePROM intervention.

Traditionally, endometriosis follow-up consisted of physician consultations at intervals of 3, 6, or 12 months by phone or in-clinic. The new telePROM-supported follow-up applied a patient-initiated and more flexible approach without prescheduled appointments for every patient (Figure 1).

With the introduction of telePROM-supported follow-up, patients could now contact the endometriosis team by asynchronous text messaging in the chat, e.g., for questions or to request a consultation. The response time for specialised endometriosis nurses was three working days.

In their appointment letter, patients were informed about the ‘Mit Sygehus’ app (version 23.2.12) and the need to complete the endometriosis-specific questionnaire prior to consultations. Technical support was provided to patients by the hospital’s IT management via telephone during weekdays in the daytime. Patients automatically received the endometriosis-specific questionnaire 14 days before their consultation through the ‘Mit Sygehus’ app, followed by an automatically generated notification. Additional information about endometriosis and instructions on completing the endometriosis-specific questionnaire were available in ‘Mit Sygehus’. The endometriosis-specific questionnaire included 54 questions about endometriosis symptoms and general well-being, and two additional free-text fields allowed patients to add further information about: (A) specific health issues relevant to the physician, and (B) the most important topics for discussion during their consultation. Patients’ responses to the endometriosis-specific questionnaire were stored in their electronic medical record.

Specialist nurses were responsible for patient triage and the endometriosis-specific questionnaire served as a screening tool to assess patients’ need for consultation. This was further supported by a severity algorithm using a green, yellow, or red colour code (Table 1).

In addition, the algorithm provided the nurses with a text summary suggesting a relevant healthcare professional and consultation mode based on the patient’s questionnaire responses. Nurses then ensured that patients were invited for an appropriate consultation with either a physician or a nurse, conducted in the clinic, via video, or by phone. The endometriosis-specific questionnaire was piloted and validated in regard to content validity and test–retest reliability by the research team prior to implementation of telePROM [30]. During consultations, the endometriosis team logged into ‘Mit Sygehus’ to use telePROM elements, including video, chat, and patients’ responses to the endometriosis-specific questionnaire [31].

The implementation of telePROM led to major changes to the clinic’s patient bookings. The team’s medical secretary was responsible for ensuring the following: that the new patient-booking principles were adopted by colleagues; for rescheduling patients triaged for a new contact; and finally, that all consultations slots were utilised at all times. Consultation slots were reserved for urgent cases as well. The endometriosis team, which included nurses, physicians, and medical secretaries, received technical and communication in situ training on using patient-reported outcome measures during consultations, with a particular focus on video consultation, because patient-reported outcome measures, video consultations, and nurse-led consultations were new to the team. The training programme has since been further developed and piloted as an innovative ap-proach of implementing video consultations in hospital settings [32].

### 2.3. Study Participants

Purposive sampling was applied to ensure participants with experience in telePROM, and we strived to establish various and relevant insights of telePROM. Therefore, the study population consisted of both physicians, nurses, and medical secretaries, all of whom formed the department’s endometriosis team (N = 11) and were involved in the daily use of the intervention. The only inclusion criterium was a minimum of three months’ experience working with telePROM. One eligible participant was on planned leave and not invited for the study.

Participants received verbal and written study information from the first author before signing a consent form including observations, informal interviews and focus group (FG) interviews. Since the first author was known to participants as the department’s specialist nurse involved in developing and implementing telePROM, it was emphasised that participation in the study was voluntary, and participants were encouraged to speak freely about their experiences. Consent was confirmed face-to-face prior to every observation, allowing participants to decline on any given day. The study was approved by the Data Protection Agency of the Region of Southern Denmark (jrn.nr 20/35407), and ethics approval was not required under Danish law.

### 2.4. Data Generation

Data were generated through observations in the endometriosis outpatient clinic between January and June 2023, in total 24 days, followed by a single observation in the outpatient clinic again in February 2024 as well as an observation of an annual team meeting and two FG interviews with members of the endometriosis team in March 2024. Data generation was carried out independently by the first author, but under guidance from co-authors AS and CN. The first author, a female registered nurse and PhD student, had prior research experience, including in qualitative methods such as interviewing.

Daytime observations in the outpatient clinic focused on healthcare professionals’ use of telePROM, e.g., including the endometriosis-specific questionnaire for triage and consultations, answering text messages in the chat, and conducting virtual consultations with patients. In addition to observing the use of technology, the daily routines of healthcare professionals and the workflow in the outpatient clinic were also observed to gain deeper insights into the culture and premises for integrating telePROM into clinical practice. First author strived to maintain minimal participation when conducting observations. Short, informal interviews were conducted between patient consultations to elaborate upon healthcare professionals’ clinical considerations and decisions regarding triage and patient treatment. These interviews were documented through descriptive field notes, and follow-up questions arose spontaneously but were driven by the research aim. A single observation was conducted in the outpatient clinic in 2024, focusing on patient triage and nurse-led chat communication to identify any new workflows that may have developed since the previous observations. At the endometriosis team’s annual meeting in March 2024, telePROM was a subject of evaluation and discussion facilitated by the hospital’s patient-reported outcome consultant and the nurse specialist from the endometriosis team. Consent for observation was confirmed by all participants attending the meeting, and observations focused on how the team discussed telePROM, its sustainability, and further development in endometriosis care. Field notes were continuously obtained during observations, and reflections were added after each observation.

FGs were conducted as they are well-suited for explorative studies—they facilitate participant discussion and elicit different viewpoints [33]. As our study aims to explore telePROM-supported follow-up and its integration in daily practice, FG discussions among team members were conducted to provide nuances, generate reflexive thoughts among participants, and identify potential new insights, along with benefits and challenges. A semi-structured focus group guide was employed during the two FGs. The guide was based on current literature, observations from the clinic, and the Model for Assessment of Telemedicine (MAST) in terms of organisation, patient safety, and utilisation of time [34]. The guide included questions such as ‘How do you find communicating with patients via the chat?’, ‘What are the benefits and challenges?’ and ‘What are your experiences of time utilisation in telePROM-supported follow-up’? A questionnaire about the participants’ age, educational background, and clinical experience with telePROM was completed prior to the interview. Each FG included five participants, mixed by profession and years of experience with telePROM, ensuring that both novice and experienced perspectives were represented. FGs were held at the beginning of the workday in a private room located in the hospital but separate from the outpatient clinic. Each session was scheduled to last an hour. The first author facilitated the FGs supported by co-author CN, who observed and wrote notes during both interviews and engaged in reflections with the first author after each interview. The interviews were audio-recorded and transcribed verbatim by the first author.

### 2.5. Data Analysis

**Figure 2:**
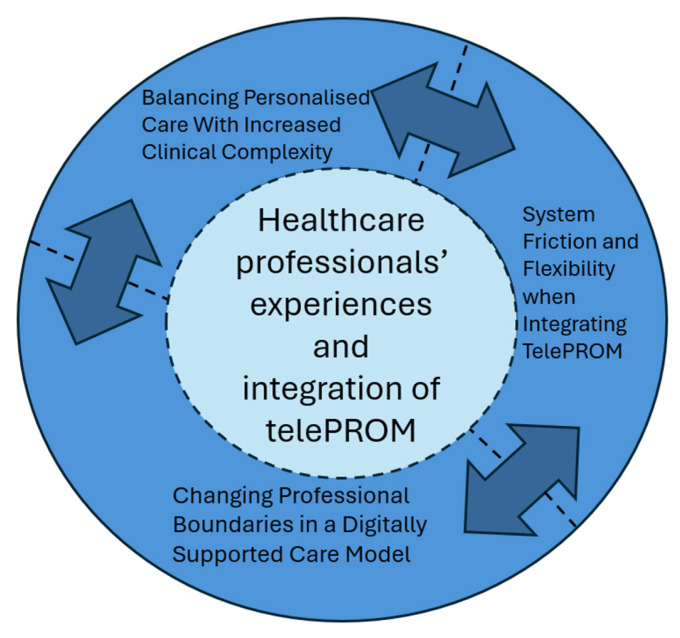
Exploration of healthcare professionals’ experiences and integration of telePROM in outpatient endometriosis care.

Transcriptions of FGs and notes from observations and informal interviews were transferred to the software programme NVivo 15 for triangulation and data analysis. During analysis, observations and interviews were weighted equally. The analysis was inductive, inspired by interpretive description, and conducted through an iterative process by the first author [27,28]. Firstly, observations and field notes provided insights into the healthcare professionals’ actions during outpatient follow-up and enabled analysis to clarify what actually happened with regard to the use and integration of telePROM. Next, the team meeting enabled analysis of current challenges and future perspectives of telePROM in endometriosis care. With observations informing the FG guide and first-author re-engaging with the same participants several times during observations, the FGs enabled analysis to further explore, expand and clarify tentative associations, including both benefits and challenges regarding telePROM’s integration in clinical care. As a first step in analysis, prior to the initial coding, a thorough reading of all observations and interview transcripts was completed to gain an overall understanding of the data set. A code was then applied to the text segments whenever they provided meaningful insights into healthcare professionals’ experiences of telePROM-supported follow-up of endometriosis, as well as their needs and challenges when integrating telePROM in clinical practice. Secondly, patterns and relations across data were searched for, codes were grouped together leading to the third step of exploration of tentative themes and primary categorization. Later and the fourth step in analysis, final themes and interpretation was formed by first-author asking the questions “And so what?” and “What does it mean?” and findings were illustrated by a figure (Figure 2) [28]. In addition, the use of reflective memos, re-engagement with participants during data generation, and ongoing discussions with co-authors AS and CN during the analysis enhanced analytical rigor and supported critical appraisal of interpretations. This process contributed to the generation of meaningful, credible findings applicable to clinical practice.

### 2.6. Rigor

To address researcher subjectivity [27], first author’s pre-understanding and reflections during data collection were written down. However, as participants were familiar with the first author, this may have influenced the results. To mitigate this, specific steps were taken during observations and focus groups, including the use of contrast questions and encouraging participants to discuss potential negative effects of telePROM. The analysis also focused on identifying challenges associated with telePROM. Throughout analysis, critical reflections were sought by asking questions such as “What am I seeing?” and “Why am I seeing that? In addition, the analysis also focused on identifying challenges associated with telePROM, supported by NVivo for data coding. Finally, the reflective memos, combined with re-engaging with participants several times and having ongoing discussions with co-authors AS and CN during the analysis, enabled analytical rigor and critical appraisal of interpretations.

## 3. Results

All eligible participants consented to being observed and interviewed (*n* = 10). The FGs lasted between 52 and 56 min. Participant characteristics are presented in Table 2.

The findings, formed by three themes, illustrated healthcare professionals’ experiences of outpatient follow-up when supported by telePROM, and their perceptions of integrating telePROM into routine clinical care: (1) Balancing Personalised Care With Increased Clinical Complexity; (2) Changing Professional Boundaries in a Digitally Supported Care Model; and (3) System Friction and Flexibility when Integrating TelePROM. In the following presentation of results, the term ‘participants’ refers to the entire endometriosis team, whereas ‘nurse’, ‘physician’, or ‘medical secretary’ denote individual professional roles.

### 3.1. Balancing Personalised Care with Increased Clinical Complexity

The participants found that telePROM enabled more personalised patient care enabling patient triage for relevant healthcare support, more focused consultations addressing current problems, and easier access to support through text messaging. The endometriosis-specific questionnaire’s algorithm was important to identify the patients with an immediate need for consultation. However, nurses gathered additional information from patients’ electronic medical record before determining the patients’ need for follow-up, e.g., whether a scan or surgery was planned or not, and from previous text messages in the chat:

“It is not just that she [the patient] only has the questionnaire. She also has the chat function. So, what you do not capture in the questionnaire, you capture through the chat [---]. One is a prerequisite for the other and vice versa.” (Participant 1)

Triage decisions were observed to vary, meaning that sometimes, patients were asked to wait and see if any symptoms would evolve, while others were given an appointment and asked to cancel via text message if their symptoms improved. The chat appeared to enable clear communication and flexibility in patient triage, when triage decisions were communicated in writing to patients through the chat. Participants expressed how they were attentive to meet patients’ expectations and clinical need in the best way possible, and their concern of patients answering untruthfully to receive an appointment was not substantiated. However, when nurses did not follow the triage recommendation provided by the algorithm—for example, when the algorithm suggested a nurse consultation, but the consultation involved surgery planning with a physician—the nurses ensured that reasons behind their triage decision were documented in the patients’ electronic medical record, thus to ensure transparency in triage decisions. Overall, it seemed that telePROM facilitated closer collaboration between patients and healthcare professionals. However, it appeared that patient triage was complex and not based alone on triage recommendations of the algorithm, demonstrating that all elements in telePROM were prerequisites for each other, supplementing one another to provide patients with relevant and improved follow-up.

The participants described how the endometriosis-specific questionnaire made participants feel better prepared for consultations with patients. Additionally, time spent with the patient was better utilised, as the endometriosis-specific questionnaire enabled healthcare professionals to focus the consultation on the patient’s individual needs rather than predefined ones, addressing areas of concern reported by the patients themselves:

“I actually think [time] is better spent than the regular consultations. [---] We are better prepared before the patient enters [consultation]. We can skip quite a bit during consultation because we are prepared [---].” (Participant 2)

In between follow-up consultations many patient issues were observed to be handled by nurses through text communication in the chat, e.g., prescription renewals or evaluation of worsening pain using the endometriosis-specific questionnaire, thereby enabling support of more patients, not needing to have an appointment with the clinic staff and seemingly optimising the endometriosis care pathway.

“I think [the chat function] is very important for [patients]. They have this lifeline [---]. They really feel seen and heard, that it is those with the greatest competencies are the ones providing answers.” (Participant 6)

Prior to the introduction of telePROM, healthcare professionals found that some patients without a clinical need were nonetheless seen in-clinic, leading healthcare professionals to feel that the time invested—both theirs and the patients’—was not meaningful. When the patients found themselves having to wait too long for a consultation, healthcare professionals viewed chat communication as a relevant offer that could further assist patients in receiving support. Furthermore, the patients were asked to send a text message to the team when their magnetic resonance imaging scan had been performed, enabling timely discussions at the multidisciplinary conference and improved fast-track and personal treatment planning. Adding video consultations as another support option instead of seeing most patients in-clinic was valued by the participants. The visual contact and observation of non-verbal cues enabled them to interact more effectively with the patients. In summary, it seemed that the healthcare professionals could better provide timelier and person-centred care for patients by using telePROM and providing patients with self-reporting their symptoms, video consultations and chat communication. Overall, it seemed to improve endometriosis care from a clinical perspective.

### 3.2. Changing Professional Boundaries in a Digitally Supported Care Model

As telePROM was integrated in follow-up, professional boundaries changed among team members. It became clear from participants’ statements that clinical experience in endometriosis was important as nurses assumed new responsibilities in telePROM, e.g., nurse-led patient triage, video consultations and support through text messaging. Nurses who were newly assigned to the endometriosis team tended to triage more patients for consultation rather than resolving issues through text-based communication in the chat, and more treatment plans required confirmation by another team member. One participant noted that an experienced nurse would answer to up to 20 messages per hour, suggesting that clinical expertise may shape not only how telePROM is implemented but also its overall effectiveness in supporting patient care.

Changes in professional boundaries caused feelings of uncertainty, particularly among less experienced staff. The participants’ reflections suggested that uncertainty was mitigated by access to support from more experienced colleagues. Being able to discuss patient cases was reported as integral towards safe decision-making and helped sustain confidence when working within the telePROM model. Rather than operating independently, regardless of profession or years of clinical experience, participants emphasised a shared sense of responsibility, where clinical specialisation in endometriosis and real-time supervision from team members functioned as crucial safeguards to maintain care quality. As one participant reflected:

“As someone who’s less experienced in the team, I always have the physician [to consult]. And we [the patient and the nurse] can just communicate in the chat afterwards. So, I always feel like I have someone I can go to if any questions arise in the video consultation.“ (Participant 3)

Team members appeared to feel comfortable discussing and reflecting on their clinical considerations in patient care and they resolved patient issues together. Thus, teamwork and shared reflection appeared valued as part of clinical decision-making, shaping the quality of care, and therefore important when using telePROM.

With telePROM nurses were predominantly those who resolved technical problems when such issues arose. Moreover, when some physicians were either unsure of or inconsistent in their active use of the endometriosis-specific questionnaire during consultation, nurses would display the patient’s endometriosis-specific questionnaire on their computer screen to support its integration during consultation. In contrast, nurses faced challenges in text message communication when correspondence extended over several days. The nurses often used everyday language and abbreviations which differed from their nurse colleagues’ wordings. Consequently, additional time was required to understand the context of the ongoing discussion between the patient and the colleague before determining how to appropriately phrase their response. TelePROM appeared to require new communicative and technical skills among participants; however, team members supported one another in managing these demands, and overall attitudes towards using telePROM during follow-up were positive. That being said, one participant did note that her personal preferences in providing patient care were challenged by telePROM:

“They [the patients] seem very happy about it [red. video consultations]. For me, it is still just that I really like having them attend physically [in the clinic]. But that is just a habit from my old nursing days [laughs].” (Participant 8)

Physicians also reported that the introduction of telePROM reshaped their clinical workload distribution by shifting routine follow-up contacts to nurses via asynchronous text communication and nurse-led video consultations. As a result, physicians reported that they spent more time consulting patients with more complex needs, which was described as making clinic work more mentally demanding.:

“We do not have those straightforward patients for consultation like we had [before]. That also makes it a tough outpatient clinic to staff. [---] But I think the time is also better spent. I think we see the patients we need to see now.” (Participant 5)

“[---] It is a need-related visits [---]. Instead of hopelessly just seeing all patients [without prioritisation].” (Participant 6)

Despite reported increased cognitive and emotional workload for physicians, they noted the telePROM-supported follow-up as appropriate and valuable, highlighting improved prioritisation and more efficient use of time and resources. So even though professional boundaries seemed to change in telePROM, including workload distribution, the close interdisciplinary collaboration seemed to provide both nurses and physicians with both professional confidence and the flexibility to manage patient consultations during their workday. Therefore, in summary, clinical experience, interdisciplinary collaboration and supervision seemed equally essential to implementing a digitally supported care model such as telePROM.

### 3.3. System Friction and Flexibility When Integrating TelePROM

The structured workflow in telePROM and the outpatient clinic was challenged by the overall organisation of physicians’ and nurses’ concurrent responsibilities in the gynaecological department. Physicians were to conduct rounds in the gynaecological ward, supervise junior colleagues throughout the day or assist with acute ultrasound examinations of admitted patients—all while also managing the endometriosis outpatient clinic. These overlapping activities sometimes caused friction so as to enable supervision when working with telePROM, e.g., during patient triage or responding to patients’ text messages. These frictions further caused delays in the outpatient clinic, which then challenged video consultations in telePROM, because they were scheduled at fixed times, leaving patients waiting online and feeling uncertain:

“Some days are just busier than others, where the physicians often are really busy doing rounds in the ward [---]. Is there time to do that if you need them for triage? I am not sure. But sometimes it is really busy. And then they [physicians} also get disturbed between consultations, where we might have 5 min to ask [about a patient case], but then they are pulled in from the ward. It happens really often.” (Participant 3)

However, the physical location of the outpatient clinic close to the gynaecological ward was important, when implementing telePROM, including the joint consultation rooms for physicians and nurses working with telePROM. The organisation of team members being closely located to each other in the hospital, also when having shifts in the gynaecological ward, seemed to enhance the integration of telePROM as team members were easy to reach when needed. Only during phone or video consultations, joint consultations rooms were replaced with separate rooms to ensure privacy. Yet, this was not always feasible due to limited availability of extra rooms. In such cases, participants were observed to switch to a phone consultation, hindering the integration of telePROM due to organisational constraints.

Additionally, system frictions appeared when the telePROM setup was challenged by new healthcare agendas within the hospital, including new technology. During the study period, patients gained the right to have a diagnostic assessment within 30 days of referral. Consequently, telePROM consultation slots were suddenly reassigned to newly referred patients, resulting in patients with red-flagged endometriosis symptoms not receiving a consultation within the designated 3, 7, or 14-day timeframe as specified in the telePROM guideline. In addition, participants were introduced to many new technologies alongside tele-PROM implementation, e.g., speech recognition, a new patient electronic medical record, and a new app for nurses’ work schedules. This made a participant comment that she was not averse to new initiatives per se, but they had to be meaningful, indicating that she was a bit overwhelmed by all the new initiatives. In addition, observations revealed that technical system faults frequently occurred in the outpatient clinic, not only limited to telePROM, and the technical set-up in the consultation room posed challenges for the use of telePROM for some participants:

“I often have it [the endometriosis-specific questionnaire] on the screen, but I have so many systems, I actually need a screen [more]. So, I think it is a bit of a problem.” (Participant 9)

Thus, it appeared that telePROM-supported follow-up was hindered by numerous system frictions and general technical issues, which challenged healthcare professionals in their daily use of telePROM, sometimes resulting in non-use of video consultations and patients’ responses to the endometriosis-specific questionnaire during consultations, and which may contribute to resistance towards its further integration and sustainability.

Still, telePROM enabled more flexibility in nurses’ daily work, which participants appreciated and which both patients and the whole department benefitted from. For example, nurses could respond to text messages remotely or while performing other duties, such as working in the gynaecological ward or in between nursing tasks. During busy periods, the flexibility in telePROM allowed the nurses to assist each other with messages as well as adjust text response times during holidays or periods of time constraints, and with 18 messages per day observed. Text communication resolved many patient issues online, prevented unnecessary consultations, and reduced interruptions for the secretary and ward, as participants experienced fewer patient phone calls as nurses now handled patient triage and patient questions through ‘Mit Sygehus’.

As familiarity with telePROM grew, participants generated new ideas that further improved telePROM-supported follow-up. The participants created automatic phrases for the most common replies in the chat, such as a referral was needed from the general practitioner to receive an appointment, and they worked to integrate telePROM further with patients’ electronic medical record. Patients with cognitive or language barriers were not eligible for telePROM-supported follow-up and were informed to call the clinic for support as they did prior to telePROM. Thus, it seemed that tele-PROM was dynamic, being adjusted and developed further by the participants to ensure its sustainability, while ensuring standard care for non-eligible patients. In summary, telePROM was challenged when shifting healthcare agendas and initiatives arose, requiring continuous attention and adaptation from dedicated personnel. Yet, enabling flexibility in work management, appeared to benefit not only nurses but the organisation as a whole and supporting efficiency in endometriosis care.

## 4. Discussion

The results demonstrate that the participants were positive towards telePROM and experienced improvements in outpatient follow-up. TelePROM enabled the patients to receive personalised support based on their current needs and through flexible virtual consultations and text communication with the endometriosis team, which also seemed to reduce unnecessary consultations as well as disturbances caused by patients’ phone calls. However, physicians found clinic work to be more mentally demanding following the introduction of telePROM, since primarily patients with complications were referred for video consultations or in-clinic visits with physicians. The analysis highlighted that clinical experience, interdisciplinary collaboration and supervision were pivotal for the effective use of telePROM. Meanwhile, shifting healthcare agendas and persistent technical issues challenged telePROM and, potentially, its integration into clinical practice.

Overall, our results indicate that a telemedicine follow-up approach, including a patient-reported outcome measure, can support healthcare professionals in providing patients with personalised, person-centred endometriosis care. Patient-reported outcome measures support patients’ perspectives during triage [14] and patient dialogue [24]. Remote monitoring of diseases is often done using patient-reported outcome measures in combination with biomarkers, such as renal disease and cancer [35,36]. However, in endometriosis no biomarkers exist, wherefore follow-up relies on patients’ symptom burden, and why patients’ self-reported data can act as a ‘biomarker’ and provide important insights about the patient.

An important finding from our study, and one that differs from traditional patient monitoring using patient-reported outcome measures, was that our setup included text communication, which enabled more nuanced information and thereby improved patient triage and increased patient safety. However, criticism has been raised that self-reported data can be influenced by mood or recall bias [37,38], and therefore the validity of the data during treatment may be questioned. Recent research in telemedicine and endometriosis explores wearables to provide objective data [38], complementing self-reported symptoms by patients. These insights may improve patient monitoring and serve as a valuable tool during outpatient follow-up, e.g., in patients with flare-ups or insufficient treatment response. Collectively, these technological advancements, including text communication, can play a role in the future monitoring and follow-up in endometriosis, providing patients with person-centred care and addressing their needs and concerns regarding endometriosis and everyday life.

However, our study also identified that physicians perceived clinic work as more mentally demanding, as they now saw only patients with severe symptoms—an observation that aligns with findings from other healthcare settings [39]. Therefore, attention must be paid to healthcare professionals’ perceived workload, mental health, and job satisfaction when introducing patient triage based upon patient-reported outcome measures. Collaboration with department managers, enabling feedback and supervision have shown to be important, when integrating telemedicine in outpatient care [32], and combined with ongoing adjustments to the outpatient clinic programme could be means to mitigate this burden, enabling physicians to not only voice their concerns but also provide input to reorganisation and adaptation to their work day in the outpatient clinic. Moreover, it has been argued that healthcare professionals may struggle to distinguish between patients at different stages of disease if care trajectories primarily consist of severely ill patients [14]. However, the increased mental workload for physicians was offset by the optimised utilisation of their time and resources.

Our study found that clinical experience, interdisciplinary collaboration, and supervision were pivotal in enabling follow-up supported by telePROM. Training of healthcare professionals is key to facilitating the digital transformation of healthcare [40], as digital care pathways require a broad range of competences. These include counselling competence related to system use and supporting patients’ self-care; information technology competence, competence in creating an interactive counselling relationship; information management, ethical competence related to digital counselling; competence in developing digital patient services; and change competence [41]. For example, integration of text-based communication in healthcare requires strong written communication skills and careful consideration of language use to ensure that patients understand the written advice provided. Yet, patients reported no difficulty understanding lengthy text messages during postpartum follow-up [42]; however, these findings may be influenced by differences in study population (e.g., younger patients) and context. An in situ training programme in video consultations piloted in a gynaecological outpatient setting demonstrated positive outcomes among patients and healthcare professionals. However, the pilot also highlighted the importance of ongoing technical support for patients and healthcare professionals, as well as the need to include patients’ preferences in patient triage [32,43]. This aligns with our findings, as not all healthcare professionals felt confident using the technology, which may contribute to limited adoption and sustainability of telePROM in clinical settings. Therefore, it is important to ensure ongoing training and supervision in digital care pathways.

As the intervention began in 2019, our results demonstrated how the telePROM setup was further developed as participants became more familiar with the technology and adopted it in their clinical practice. Our result illustrates how technology is neither neutral nor fixed when being implemented in clinical practice, supported and explained by the Actor-Network Theory, highlighting how telemedicine and social practice interact and co-evolve [44]. TelePROM encompasses a complex network of interactions between human actors, including the endometriosis team, managers, IT supporters, and non-human actors such as ‘Mit Sygehus’, the endometriosis-specific questionnaire, guidelines, Wi-Fi, legislation (General Data Protection Regulation). All are equally important and needed to assemble, align, and finally stabilise the network [44,45,46]. Yet, stabilising telePROM requires training, adaptation, and changes to routines [44]. In this study, this was sought met by in situ training of healthcare professionals in the use of the endometriosis-specific questionnaire during video consultations, development of patient pathway guidelines, as well as support by management and IT. But telePROM-supported follow-up can be challenged and destabilised, not only by organisational changes but also by technology and humans, and therefore, telemedicine interventions require continuous attention and adaptation to be used in clinical practice. Our findings disclose the need to rethink the setup of telemedicine and IT support, focusing on on-site support, which is essential during the digital transformation of healthcare. Potential barriers in healthcare organisations could be lack of resources and technically challenged healthcare professionals [47]. Yet, key local stakeholders in telemedicine can support, stabilise, and sustain new healthcare technologies in hospital settings, potentially increasing healthcare professionals’ digital skills and reducing technical disruptions. In our study, nurses seemed to have taken on the role of providing technical support regarding telePROM, which supported the initiative’s sustainability. The use of telemedicine in general, including patient-reported outcome measures, has been suggested to have a negative impact on the clinical workload and workflow [48]. However, this was outweighed by the optimised utilisation of time and resources among our participants. Integrating asynchronous text messaging in follow-up could lead to an increasing number of patient contacts, but these interactions are often shorter and can reduce the need for traditional in-clinic visits. That being said, more studies of telePROM are needed to evaluate the impact on healthcare professionals’ time, which is rarely researched in telemedicine [49].

Another key finding was the importance of the physical location of the outpatient clinic, which was closely connected to the ward and medical secretaries. However, this proximity also led to interruptions, such as junior physicians requiring supervision or admitted patients needing acute ultrasound examinations. When implementing telemedicine and patient-reported outcome measures, considerations related to supervision and patient safety are therefore important. Patients’ experiences with telePROM highlighted a need for a patient education programme to support engagement [24] and strengthen patient safety. Likewise, thorough introduction, on-going supervision, and training are essential among healthcare professionals when organising and planning remote monitoring and follow-up, with attention to disease-specific considerations. By exploring the perspectives of both patients and healthcare professionals, a holistic and complementary approach for further improvement of digitally supported healthcare in endometriosis is provided.

### Strengths and Limitations

Our study has limitations, as this was a single-centre study with a small sample size (*n* = 10). Although supported by participants’ observations in the clinic, this may still have implications regarding the transferability of the results to other settings. However, novel innovative telemedicine solutions in healthcare need to be evaluated to ensure patient and clinician compliance, as well as safety and quality of care before generalisations can be made and potential upscaling considered. Variation in participants and disciplines was intentionally included to gain broad and nuanced insights into telePROM. Further investigation of telePROM is necessary and should include clinical outcomes to fully explore telePROM’s potential in clinical practice and facilitate its implementation in other endometriosis care settings.

The strength of this study was that the telePROM setup was co-developed with patients and the endometriosis team. This ensured that telePROM was person-centred and user-friendly by addressing the needs of both patients and healthcare professionals, thereby facilitating the implementation of the intervention. However, because the implementation of telePROM was self-imposed by the endometriosis team and departmental management, this could potentially affect participants’ experiences of telePROM’s influence on outpatient follow-up. Still, the participants’ initial enthusiasm appeared not to have driven and influenced the results of this study, as the intervention began in 2019 with the introduction of the endometriosis-specific questionnaire and video consultations in the outpatient clinic and reflects several years of healthcare professionals’ experiences with the intervention. The Danish digital infrastructure, including a regional app poses a strength for transferability and further adaptation to other healthcare settings.

## 5. Conclusions

Telemedicine in outpatient endometriosis care was found to be relevant, as it optimised patient care. TelePROM enabled personalised follow-up through flexible support options and supported by patients’ questionnaire responses, thereby seemingly preventing unnecessary consultations. However, to integrate telemedicine safely in routine outpatient follow-up, it appeared essential to ensure clinical experience with the disease and its treatment options, interdisciplinary collaboration and supervision. Moreover, text communication provided additional patient information that was important for patient triage.

The interactions among humans, telemedicine, and organisations change over time. Therefore, it is important to ensure ongoing training of healthcare professionals to develop competencies in digital care pathways. Organisational resilience may need to be strengthened during the digital transformation of healthcare, which may require new ways of organising support options for digital care. These initiatives may facilitate and influence the broader integration of telemedicine within healthcare systems.

## Figures and Tables

**Table 1:** Simplified outline of the endometriosis-specific questionnaire algorithm.

Colour Code		Description	Summary
Red	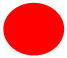	Significant problem	In-clinic consultation by physician
Yellow	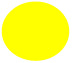	Mild to moderate problem	Video or phone consultation by nurse or physician
Green	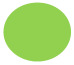	No problem	No need for consultation

**Table 2:** Study participants’ profile (*n* = 10).

Age Range	27–67
Profession	
Physician	4
Nurse	5
Medical Secretary	1
Gender	
Male	1
Female	9
Clinical experience with telePROM	
≤1 year	1
>1–≤3 years	5
>3 years–≤5 years	4

## Data Availability

The participants of this study did not consent to public sharing of their data. Due to the sensitive nature of the research and the potential identifiability of the sample, supporting data are not available.

## Abbreviations

The following abbreviations are used in this manuscript:

TelePROM	Tele-Patient-Reported Outcome Measures
